# MELTING, a flexible platform to predict the melting temperatures of nucleic acids

**DOI:** 10.1186/1471-2105-13-101

**Published:** 2012-05-16

**Authors:** Marine Dumousseau, Nicolas Rodriguez, Nick Juty, Nicolas Le Novère

**Affiliations:** 1 EMBL-EBI, Wellcome-Trust Genome Campus, Hinxton CB10 1SD, UK

## Abstract

**Background:**

Computing accurate nucleic acid melting temperatures has become a crucial step for the efficiency and the optimisation of numerous molecular biology techniques such as *in situ* hybridization, PCR, antigene targeting, and microarrays. MELTING is a free open source software which computes the enthalpy, entropy and melting temperature of nucleic acids. MELTING 4.2 was able to handle several types of hybridization such as DNA/DNA, RNA/RNA, DNA/RNA and provided corrections to melting temperatures due to the presence of sodium. The program can use either an approximative approach or a more accurate Nearest-Neighbor approach.

**Results:**

Two new versions of the MELTING software have been released. MELTING 4.3 is a direct update of version 4.2, integrating newly available thermodynamic parameters for inosine, a modified adenine base with an universal base capacity, and incorporates a correction for magnesium. MELTING 5 is a complete reimplementation which allows much greater flexibility and extensibility. It incorporates all the thermodynamic parameters and corrections provided in MELTING 4.x and introduces a large set of thermodynamic formulae and parameters, to facilitate the calculation of melting temperatures for perfectly matching sequences, mismatches, bulge loops, CNG repeats, dangling ends, inosines, locked nucleic acids, 2-hydroxyadenines and azobenzenes. It also includes temperature corrections for monovalent ions (sodium, potassium, Tris), magnesium ions and commonly used denaturing agents such as formamide and DMSO.

**Conclusions:**

MELTING is a useful and very flexible tool for predicting melting temperatures using approximative formulae or Nearest-Neighbor approaches, where one can select different sets of Nearest-Neighbor parameters, corrections and formulae. Both versions are freely available at http://sourceforge.net/projects/melting/and at http://www.ebi.ac.uk/compneur-srv/melting/under the terms of the GPL license.

## Background

The quality of many experiments in molecular biology depends on the accurate characterisation of the helix-coil transition of nucleic acid duplexes. This includes for instance Polymerase Chain Reaction (PCR) experiments, sequencing by hybridization, antigene targeting, southern blotting, prediction of local stability within a duplex, and predicting the influence of mutations on duplex stability. For some of these applications, being able to predict accurately the melting temperature avoids the amplification or detection of wrong sequences.

In addition, it has been established that RNA molecules, besides a role in encoding proteins, also have more complex functions, for instance as microRNAs and siRNAs. These small non-coding RNA molecules are involved in the regulation of gene expression by means of direct RNA-RNA binding. These discoveries have driven the need for algorithms and software providing accurate predictions for hybridization, melting temperature, DNA/RNA folding and secondary structures. A lot of these needs are already covered by tools such as The Vienna RNA WebServers [1], which implement a large set of algorithms for RNA/DNA secondary structures prediction and base pair probabilities such as RNAfold or RNAcofold. More recently, the RIP program [2] and the piRNA program [3] have been developed to compute partition function over interaction complexes of RNA pairs for determining accurate base pair probabilities.

However, one of the most frequent approaches used for predicting melting temperature is the Nearest-Neighbor approach, based on the assumption that the interaction between Watson-Crick base pairs depends on the neighboring base pairs. The enthalpy and entropy for the hybridization of two complementary sequences can be simulated by the initial attachment of the first Watson-Crick base pair independently from neighboring base pairs and then its lateral propagation taking into consideration both the energy of formation of the Watson-Crick base pairs and the stacking interaction between the neighboring base pairs [4]. Two duplexes with the same base pairs could have different stabilities depending on the nearest neighbor pairs composition. However, two duplexes with different sequences but identical sets of nearest neighbor base pairs have almost identical thermodynamics properties [5]. The formula for the melting temperature depends on the oligonucleotide concentration, the enthalpy (*δh*) and the entropy (*δs*) of the duplex. The entropy and enthalpy values for each nearest neighbor pair which consists of two Watson-Crick base pairs have been studied for many years and several databases of thermodynamic parameters are now available such as the thermodynamic parameters for DNA/DNA hybridization and folding from Santalucia and Donald [6] or the NNDB database for RNA/RNA hybridization and folding from Turner and Mathews [7]. Several software using the Nearest-Neighbor approach and thermodynamic parameters already exist, such as DINAMelt [8], which not only compute the melting temperature of nucleic acids but also provides entire equilibrium melting profiles as a function of temperature.

However, most software computing the melting temperature using the Nearest-Neighbor method rely on a limited set of thermodynamic parameters and patterns, typically from a single author. MELTING is a free, open source software, available since 1997, which computes the melting temperature of nucleic acid duplexes using a wide set of thermodynamic datasets and formulae from the literature. MELTING does not predict duplex formation, but predicts its melting temperature for a given alignment of the duplex strands (including mismatches, internal loops, dangling ends, etc). Version 4.2 has already been integrated into several platforms such as OligoDB [9], SOL [10], SEPON [11], SiDE [12] and the siRNA selector [13]. MELTING 4.2 supports DNA/DNA, RNA/RNA and DNA/RNA hybridizations, DNA internal single base mismatches and DNA single base dangling ends [14].

As the calorimetric measurement for Nearest Neighbor parameters are made on small oligonucleotides, and the linear combination has only been demonstrated for oligonucleotides of less than 15 bases, the nearest-neighbor method cannot be accurately applied to long sequences. The complex secondary structures formed by longer oligonucleotides are more accurately taken into account by hybridization ensembles or partition functions. As MELTING does not use such methods, approximative formulae for DNA/DNA, RNA/RNA and DNA/RNA duplexes, such as the one from Wetmur [4] or from von Ahsen *et al.*[15] are used when dealing with long sequences. These depend on GC composition, duplex length and sodium concentration.

A lot of progress has been made in the field of nucleic acid thermodynamics, and parameters are available for other modified nucleic acids or biochemical molecules. These include locked nucleic acids [16], azobenzene [17] and inosine, a modified adenine base frequently used in PCR because of its universal base capacity [18,19]. Some approximative temperature corrections already exist for denaturing agents such as formamide and DMSO, both of which are frequently used in molecular biology experiments [15,20]. Similarly, corrections for the magnesium ions are also available [21-23]; these ions are frequently present in PCR experiments to activate enzymes and influence duplex stability. Oligonucleotide sequences are not always perfectly complementary and the duplex can contain mismatches, bulge loops or dangling ends. For each of these patterns, several thermodynamic formulae exist to compute resultant enthalpy and entropy. These formulae are often different according to the type of hybridization, such as DNA/DNA or RNA/RNA.

MELTING 4.x could only support a single thermodynamic formula for computing duplex entropy and enthalpy, and was unable to incorporate thermodynamic parameters for inosine nor accomodate corrections for the magnesium ions. MELTING 5 is a complete reimplementation supporting a larger set of parameters and thermodynamic formulae to compute the enthalpy and entropy of several duplex patterns such as mismatches, bulge loops, dangling ends, CNG repeats, modified nucleic acids and to include new ion and denaturing agent corrections.

## Implementation

The structure of MELTING 4 has been described elsewhere [14], and we will focus here on MELTING 5.

### Nearest-Neighbor approach

#### Algorithm

Each duplex strand is entered as a linear sequence of nucleic acids, with gaps in the sequences represented by a dash (’-’), e.g. *AATT*−−*GC*−*TA*(containing three gaps). For perfect matching, only one strand is necessary as the complementary sequence can be deduced by the program.

The Nearest-Neighbor implementation consists of several steps. First, the program determines the location of each mutually exclusive pattern (e.g. perfectly matching base pairs, mismatches, bulge loop, internal loop, dangling ends, modified nucleic acids) composing the duplex. Patterns consisting of consecutive perfectly matching Watson-Crick base pairs are located and identified first. Each remaining set of consecutive base pairs in the duplex (possibly containing gaps) are identified as requiring specific thermodynamic formulae and parameters. For example, if a base pair contains an inosine and it is adjacent to a mismatch, it will be considered as a pattern composed of two base pairs (one with inosine and another with a mismatch) requiring a formula and parameters of its own.

Once the locations of the patterns are determined, the program is able to match the pattern to its thermodynamic formula using hardcoded recognition methods, and applies the appropriate parameters to compute its enthalpy and entropy. The default formula and parameters used can be overriden by a user defined set through the options. For example, a pattern consisting of *L* perfectly matching Watson-Crick base pairs starting at position *i* in the duplex would use this formula:

(1) $$\begin{array}{cc} \Delta & H_{\text{perfectly matching pattern (i..i+L)}}\\ =\overset{L}{\underset{i}{\sum }}\delta h_{\text{nearest neighbor base pairs}}\\ \Delta & S_{\text{perfectly matching pattern (i..i+L)}}\\ =\overset{L}{\underset{i}{\sum }}\delta s_{\text{nearest neighbor base pairs}} \end{array}$$

Some patterns also take into consideration the base pairs located outside of the pattern; an internal loop pattern starting at the position *i* and ending at the position *i+L* will take into account the base pairs at *i-1* and *i+L+1* because the neighbors of the internal loop are important in determining the enthalpy and entropy. Supposing we apply the thermodynamic rules for RNA from Lu *et al.*[24], the internal loop will be computed as follows:

(2) $$\begin{array}{cc} \Delta H_{\text{internal loop (i..i+L)}} & =\delta h_{\text{initiation-loop(L)}}\\ \;+\delta h_{\text{per-adjacent-AU-or-GU}}\\ \;+\delta h_{\text{first-non-canonical-pairs}}\\ \;+(L1-L2)\delta h_{\text{asymmetry}} \end{array}$$

(3) $$\Delta S_{\text{internal loop (i..i+L)}}=0$$

Where: 

· *δh*_initiation-loop(L)_accounts for the internal loop of L nucleotides.

· *δh*_asymmetry_accounts for the internal loop asymmetry (when there is an unequal numbers of nucleotides on each side) with L1 and L2 the number of nucleotides on each strand per-adjacent-AU-or-GU accounts for each AU or GU base pair adjacent to the internal loop.

· *δh*_first-non-canonical-pairs_accounts for each sequence specific first mismatch (bonus).

This mechanism is the same for other mismatches, bulge loops and modified nucleic acids.

Before applying each thermodynamic formula, the program checks if it is applicable within the environment set by the user (hybridization type, ion concentrations). Therefore, the previous rules from Lu [24] will not be applied when the internal loop is not a 1 x (n-1) internal loop with n > 2 or when the hybridization type is DNA/DNA.

**Figure 1:**
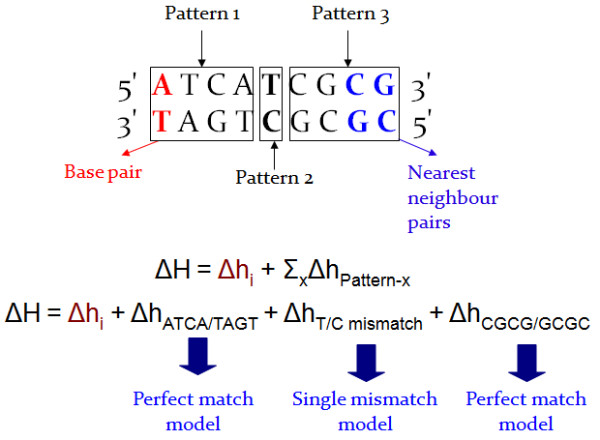
**Nearest-neighbor computation example.** The duplex is composed of 3 patterns: two perfectly matching patterns (patterns 1 and 3) and one single mismatch (pattern 2). To compute the enthalpy and entropy of the perfectly matching patterns, MELTING 5 uses a single thermodynamic formula based on user-defined or default thermodynamic parameters. Other thermodynamic formula and parameters are used to compute the enthalpy and entropy of pattern 2.

To be able to use the appropriate thermodynamic formula for each pattern composing the duplex, the program computes the enthalpy and entropy of the duplex as follows (Figure 1 is an example of Nearest-Neighbor computation):

(4) $$\begin{array}{cc} \mathrm{\Delta H} & =\delta h_{\text{initiation}}+\sum \delta h_{\text{pattern}}\\ \mathrm{\Delta S} & =\delta s_{\text{initiation}}+\sum \delta s_{\text{pattern}} \end{array}$$

The program computes the initiation of the hybridization according to the formula and parameters chosen by the user for perfectly matching sequences. It is important to note that in MELTING 5, the user selects not only a set of thermodynamic parameters (as in MELTING 4) but also the thermodynamic formula that uses it. The default set of parameters for each formula is defined by the paper describing that formula.

Finally, the melting temperature is computed using the same formula as MELTING 4:

(5) $$\mathrm{Tm}(\text{°}C)=\frac{\mathrm{\Delta H}}{\mathrm{\Delta S}+R\ln(C_{T}/F)}-273.15$$

where: 

· Tm (°C) represents the melting temperature in a solution of 1 M of sodium ion.

· *C*_*T*_is the total concentration of oligonucleotides.

· F is 1 when oligonucleotides are self-complementary. When they are not, F is 4 if both strands are present in equivalent amount and 1 if one strand is in excess (e.g. in PCR experiments).

When oligonucleotides are not self complementary, the term *C*_*T*_/*F* is replaced with *C*_max_−*C*_min_/2, where *C*_max_ is the concentration of the strand in excess and *C*_min_the concentration of the other strand. But if the excess is large enough, the total concentration of oligonucleotides can be assumed to be identical to the concentration of the strand in excess. The actual formula is $\ln(C_{\text{max}}-C_{\text{min}}/2)$ but with *C*_max_>>*C*_min_, *C*_max_ is equivalent to *C*_*T*_and $\ln(C_{\text{max}}-C_{\text{min}}/2)$ is equivalent to $\ln(C_{T})$ and so F is 1. If the excess is not important enough to make the previous assumption, we can assume that *C*_max_ is close to *C*_min_. Given that *C*_*T*_=*C*_min_ + *C*_max_, then $\ln(C_{\text{max}}-C_{\text{min}}/2)$ is equivalent to $\ln(C_{T}/4)$ which comes back to the default correction of F=4 when both strands are in equivalent amount.

When MELTING 5 initially parses the sequences, it identifies the known base pairs and stores them as a List. The program currently recognises the chemical entities with the following string representations: A (adenosine), T (thymidine), C (cytosine), G (guanosine), U (uridine), A* (2-hydroxyadenosine), Al (locked adenosine), Cl (locked cytosine), Gl (locked guanosine), Tl (locked thymidine), I (inosine), X_C (cis azobenzene) and X_T (trans azobenzene). It uses a greedy match to parse the nucleic acid names in order to solve ambiguities (e.g. A* will take precedence over A).

#### Special case of CNG repeats

The thermodynamic parameters for CNG repeats from Magdalena *et al.*[25] are applicable to self complementary sequences entirely composed of 2 to 7 CNG repeats, starting with a GC base pair and ending with a CG base pair. That the initiation is already included in the thermodynamic parameters so no additional initiation is computed for these sequences. For sequences composed of 5 to 7 CNG repeats, a hairpin pattern is dominant and the melting temperature is computed as follow:

(6) $$\mathrm{Tm}=\frac{\mathrm{\Delta H}}{\mathrm{\Delta S}}-273.15$$

#### Ion and denaturing agent corrections

The concentrations of various cations, such as sodium, and certain denaturing agents can be entered; MELTING can adjust the computed melting temperature taking into consideration the concentration of monovalent cations (sodium, potassium, Tris), bivalent cations (magnesium) and denaturing agents such as formamide and DMSO, using published formulae.

By default MELTING 5 loads the appropriate ion correction using the algorithm from Owczarzy *et al.*[21], similarly to MELTING 4.3. The ion correction to apply can be forced by the user, thereby ignoring the default choice. The purpose of this implementation is to take into account the possible binding competition between monovalent and bivalent cations by default, yet still provide flexibility should the user prefer to apply a specific ion correction. As MELTING 5 implements several sodium equivalence formulae (see [21] and [15] for more information), it is now possible to enter potassium, magnesium and Tris buffer concentrations even when an approximative computation is used, or a sodium correction is forced. This enables sodium-dependent approximative computation methods to take into account ions other than sodium.

According to Santalucia and Hicks [6], the terminal mismatches in internal loops are assumed to have the same salt dependence as complementary base pairs, whereas the stability of the remaining internal loop nucleotides are assumed to be salt independent. Consequently, MELTING 5 computes separately a ’sodium independent’ entropy and a ’sodium dependent’ entropy when internal loops of type *n*1×*n*2 are present, where n1 > 2 is the number of nucleotides in the internal loop for the first sequence and n2 > 2 is the number of nucleotides in the internal loop for the second sequence. The ion correction will only be applied to the ’sodium dependent’ entropy, and the final entropy used to compute the melting temperature will be the sum of the ’sodium independent’ entropy and the corrected ’sodium dependent’ entropy. In this situation, MELTING 5 considers the entropy term for the loop length and the entropy term for the loop asymmetry (See the internal loop formulae from Santalucia and Hicks [6]) as sodium independent.

### Approximative approach

Like its previous versions, MELTING 5 has a customisable threshold value for the maximum oligomer length for which a Nearest-Neighbor approach should be used instead of an approximative formula. It is possible to bypass this threshold and force the usage of a specific type of computation.

The software offers several approximative formulae taken from the literature such as that of Wetmur [4] for DNA:

(7) $$\begin{array}{cc} \mathrm{Tm} & =81.5+16.6\log\frac{\left[{\text{Na}}^{+}\right]}{1+0.7\left[{\text{Na}}^{+}\right]}+0.41\mathrm{\%GC}\\ \;-\frac{500}{\mathrm{size}}-\mathrm{\%Mismatching} \end{array}$$

Similar to the Nearest-Neighbor approach, the approximative formula will be used if the environment matches the condition of application. As these formulae embed sodium correction, MELTING does not apply any additional ion correction. However, if the environment contains magnesium or other monovalent cations, the approximate formula will use the equivalent sodium concentration computed by a sodium-equivalence formula such as that of von Ahsen *et al.*[15]:

(8) $$\begin{array}{cc} \left[{\text{NaEq}}^{+}\right] & =\left[{\text{Na}}^{+}\right]+\left[{\text{K}}^{+}\right]+\frac{\left[{\text{Tris}}^{+}\right]}{2}\\ \;+3.79\sqrt{\left[{\text{Mg}}^{2+}\right]-[\text{dNTP}]} \end{array}$$

### Software design

#### User interface

Upon reading the options set by the user, the chosen thermodynamic formulae, approximative formulae and ion/denaturing agent corrections are instantiated using the Factory design pattern.

There are several groups of options in MELTING 5. The *information options* display information about the program, such as help or legal information. To be able to compute a melting temperature, the program requires *mandatory options*: type of hybridization, sequence(s) and nucleic acid concentration, and at least one ion concentration. Moreover, the *general options* allow one to define the optional environment of the program: other ion or denaturing agent concentration(s), verbose mode, file output mode, data file path or threshold value. Finally, there are several options which enable the user to change the default Nearest-Neighbor formulae and/or parameters, the default approximative formulae, the ion and denaturing agent corrections or to force a specific melting temperature computation method (Nearest-Neighbor approach or approximative formulae). After the options are entered, the program first looks for the hybridization type to load the appropriate default set of thermodynamic parameters. Then the set of options entered by the user are completed with the default values for this hybridization type.

The command line option syntax of MELTING 5 differs from the previous versions. While an alternative backwards-compatible executable is provided to allow the use of the previous option syntax with the new software, it precludes use of the new features when using the old syntax.

The program automatically detects when the sequences are self complementary and sets the nucleic acid factor correction F to 1. It also applies an additional symmetry correction parameter which is stored with its nearest neighbor pairs parameters set.

### Extension interfaces

We have defined four interfaces, one for each kind of model that is used by MELTING: 

· melting temperature computation model (approximative or Nearest-Neighbor approach)

· enthalpy and entropy computation method for patterns forming the duplex

· ion and denaturing agent correction

· sodium equivalence computation method

#### Software structure

MELTING 5 is entirely written in Java and can be run with a Java 5 environment or higher. Executables for Microsoft Windows and Linux, as well as the pre-generated JAR file, are provided together with the source code. The program is licensed under the General Public License version 2 [26]. To date MELTING 5 does not have a graphical interface, but can be easily used through the command-line. The distribution contains a detailed user guide, which describes each Nearest-Neighbor or approximative formula, ion and denaturing agent correction. Detailed documentation for the developers, including a pre-generated Javadoc is also provided. The package can be found at the following addresses: http://sourceforge.net/projects/melting and http://www.ebi.ac.uk/compneur-srv/melting/.

#### Data encoding

All the sets of thermodynamic parameters are taken – or deduced – from published experimental work. Datasets are stored in XML files which allow more flexibility and extensibility for data representation (Table 1 presents the list of existing elements and attributes). In order to correspond to the literature of the domain, the enthalpy and entropy values of each parameter are given in cal/mol despite this unit not being SI compliant.

**Table 1:** MELTING 5 XML elements and attributes

Element	Attribute(s)
data	type
neighbor	sequence
initiation	type
terminal	type
symmetry	
mismatch	sequence, size, type, loop, closing
asymmetry	
penalty	type
parameters	sequence
bulge	size, sequence, type
closure	type
modified	sequence, type, sens
CNG	sequence, repeats
dangling	sequence, sens
enthalpy	
entropy	

Types of XML nodes and attributes found in thermodynamic files, defining enthalpy and entropy parameters in cal/mol.

**Figure 2:**
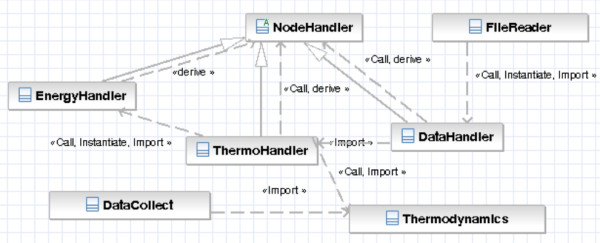
**UML diagram for XML parsing.***FileReader* calls the *DataHandler* to parse the node ’data’. *DataHandler* delegates the ’data’ subnodes parsing to *ThermoHandler* which delegates the ’enthalpy’ and ’entropy’ parsing to *EnergyHandler*. For each type of thermodynamic parameter, a *Thermodynamics* object containing the matching enthalpy and entropy values are created and stored into a map. The manipulation of the data goes through a DataCollect object.

MELTING uses SAX to parse the XML files, with an additional layer of XML-independent Handler classes to hide the XML parsing process (Figure 2 presents the UML diagram of these classes). Once sets of pairs of entropy and enthalpy values are loaded from their file, they are independent from their XML representation.

## Results

### Common new features of MELTING 4.3 and MELTING 5

In addition to the features described below, some inaccuracies have been fixed in the calorimetric tables, which explains some of the differences between MELTING 4.3 and MELTING 4.2 results.

#### Inosine parameters

MELTING 4.3 integrates thermodynamic parameters for inosine. For DNA/DNA duplexes, the program uses the parameters of Watkins and Santalucia [18], which covers all possible nearest neighbor pairs containing a single inosine base, and several nearest neighbor pairs containing two inosine bases. For RNA/RNA duplexes, the program uses the parameters of Wright *et al.*[19] which covers only Inosine-Uracil(IU) base pairing.

#### Corrections for ion concentrations

It is now possible to enter the concentration of potassium ions, Tris buffer and magnesium ions. If only a sodium ion concentration is entered, MELTING 4.3 uses a sodium correction (default correction or user specified). Otherwise, the program uses the algorithm from Owczarzy [21] to load the appropriate ion correction: one of the sodium corrections if magnesium ion concentrations are negligible, a mixed monovalent/bivalent ion correction if there is a binding competition between monovalent and bivalent cations, and a magnesium correction if monovalent cation concentrations are negligible.

### New features of MELTING 5

#### Approximative formulae

MELTING 5 provides a large set of approximative formulae for DNA/DNA, RNA/RNA and DNA/RNA duplexes from von Ahsen *et al.*, 2001 [15] that do not take into account mismatches, bulge loops and dangling ends. In addition it still provides formulae from Wetmur, 1991 [4], as implemented in previous versions, which include a penalty based on percentage of mismatch.

#### Nearest-Neighbor formulae and parameters

MELTING 5 supports several patterns in DNA/DNA and RNA/RNA duplexes: perfectly matching base pair, single mismatch, asymmetric and symmetric tandem mismatch, asymmetric and symmetric internal loop, single and long bulge loop, single dangling end, second dangling end, long poly-A queue and sequences composed of CNG repeats (Additional file 1 matches the implementation of thermodynamic formulae with their reference papers). Due to the flexibility and modularity of MELTING 5, it is now possible to choose a combination of thermodynamic formulae and parameters to compute the enthalpy and entropy of the different patterns composing the duplex. In addition to the choice of the thermodynamic formula(s), MELTING 5 allows the user to specify custom thermodynamic parameters.

However, in order to recognise patterns, MELTING 5 requires that users explicitely identify dangling ends, asymmetric internal loops or bulge loops by filling the gaps in the complementary sequence with dash characters.

For 2’-O-methyl RNA/RNA and DNA/RNA duplexes, only thermodynamic parameters for perfectly matching sequences are currently available, but the extensibility of MELTING 5 will facilitate the inclusion of these parameters in the future. As the 2’-O-methyl RNA/RNA parameters are determined in a solution of 0.1 M of sodium ions, MELTING 5 automatically applies the entropy correction from Santalucia and Hicks, 2004 [6] to each 2’-O-methyl RNA/RNA parameter before using them in the Nearest-Neighbor algorithm.

The parameters for inosine are the same as for MELTING 4.3. Parameters for other biochemical entities (see implementation section for details) are only implemented for DNA/DNA duplexes. The independence of the nucleic acid name will facilitate the integration of additional nucleic acids and fluorophores.

#### Corrections for ion concentrations

MELTING 5 accepts user defined concentrations for sodium, potassium, magnesium, dNTP and Tris buffer. The program provides several sodium, magnesium and mixed monovalent/bivalent ion corrections (Additional file 2 presents the references of all ion correction implementations).

To test the accuracy of the ion corrections as implemented in MELTING 5, the sodium corrections were compared using 66 different DNA perfectly complementary sequences from Owczarzy *et al.* (2004) [27] and five sodium concentrations (0.069 M, 0.119 M, 0.220 M, 0.621 M and 1 M) for each of the DNA sequences. To test the magnesium corrections, 18 different DNA perfectly complementary sequences from Owczarzy *et al.* (2004) [27] and five magnesium concentrations (0.0005 M, 0.0015 M, 0.003 M, 0.01 M and 0.02 M) were used. The oligomer concentration was 0.000002 M in all cases. The error margin was computed as follow:

(9) $$\mathrm{Err}=\left|\frac{\mathrm{Tm}(\mathrm{computed})-\mathrm{Tm}(\mathrm{experimental})}{\mathrm{Tm}(\mathrm{experimental})}\right|$$

Table 2 presents these results.

**Table 2:** MELTING 5 error margin per ion correction

Sodium corrections	
Sodium correction formula	Error percentage
ash01 [15]	16.4
kam71 [28]	7.1
marschdot [28,29]	9.2
owc1904 [27]	4.0
owc2004 [27]	2.3
owc2104 [27]	5.2
owc2204 [27]	2.8
san96 [30]	2.8
san04 [6]	2.2
schlif [31]	6.4
tanna06 [32]	2.9
wet91 [4]	3.0
Magnesium corrections	
Magnesium correction formula	Error percentage
owcmg08 [21]	2.16
tanmg06 [32]	4.46

Percentage of error between computed and experimental melting temperature for each MELTING~5 sodium and magnesium ion correction implementation.

**Figure 3:**
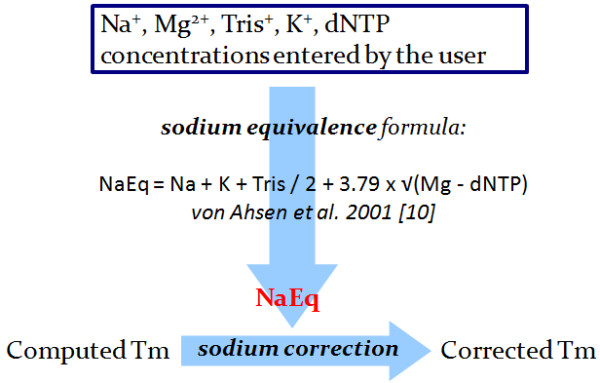
**Sodium equivalence computation.** MELTING uses a default formula to compute a sodium equivalent concentration (this figure illustrates an example of the formula from von Ahsen *et al.*[15]). This sodium equivalent concentration is then used by the sodium correction formula to modify the melting temperature calculated. This sodium correction can be included into approximative formulae or can be a separate formula applied during the Nearest-Neighbor computation. Users can overide the default formula and enter values for sodium, potassium or magnesium ion species.

Sodium equivalence computation is automatically applied on approximative formulae if several cations concentrations are present. The computed sodium equivalent concentration replaces the actual ion concentrations. During a Nearest-Neighbor computation, a sodium equivalent concentration can be computed if the user enters a specific sodium correction as well as several cation concentrations (Figure 3).

#### Denaturing agent corrections

MELTING 5 provides several approximative corrections for DMSO [15] and formamide [20]. However, these corrections are independent of the ion correction, although denaturing agents and ions interact with each other. MELTING 5 does not integrate an algorithm to take this interaction into account since none has been published. However, it is now easier to add new algorithms and denaturing agent corrections due to the flexibility and extensibility of the modular program core.

#### Self-complementarity and complementary sequence

As in the previous versions, MELTING 5 can derive a complementary sequence when only one sequence is provided. In addition, the program can automatically detect when a sequence is self complementary and applies a correction for the symmetry, as well as setting the correction factor F to 1. This automatic detection is applicable for perfectly matching sequences with and without dangling ends. If a self complementary sequence containing mismatches, modified nucleic acids or bulge loops is to be entered, only the first sequence should be entered, with the new option *-self *, which will inform MELTING of the self-complementarity.

#### Type of hybridization

MELTING 5 requires that users enter a type of hybridization since this affects the thermodynamic formulae and parameters used. When the user enters the hybridization type, MELTING 5 is now sensitive to the order of the sequences types. Indeed, a *dnarna* hybridization indicates that the 5’3’ sequence entered with the option -S is a DNA sequence and the 3’5’ sequence entered with the option -C a RNA sequence. A *rnadna* hybridization would indicate the opposite.

## Discussion

Since the software architecture of MELTING 4 was able support the addition of inosine parameters and magnesium ion correction, MELTING 4.3 was released to allow software already embedding MELTING 4 to be easily updated and benefit from these important new features. However, the limitations of the architecture of MELTING 4 did not allow the incorporation of new thermodynamic parameters and formulae for patterns other than perfectly matched sequences or single base mismatches. This necessitated a complete rewrite of the software which now includes new features such as DMSO and formamide corrections, internal loop parameters, bulge loops, initiation parameters depending on the base pairs and modified nucleic acids. MELTING 4 is now superseded by MELTING 5 and users are invited to upgrade at their convenience. However, unlike MELTING 4, a graphical interface has not yet been implemented for MELTING 5.

### Accuracy of the predictions and flexibility of MELTING 5

**Figure 4:**
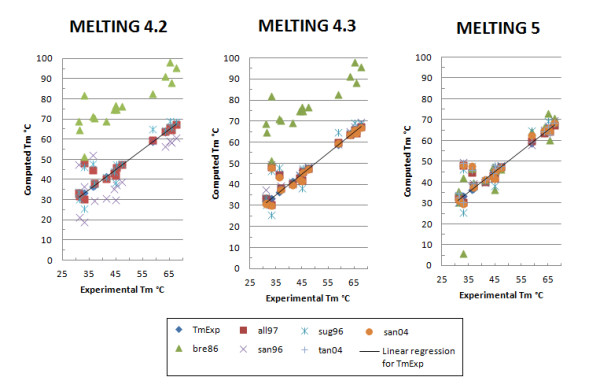
**MELTING results for DNA sequences.** The parameters sets specified by Allawi *et al.*[33] (all97), Breslauer *et al.*[34] (bre86), SantaLucia *et al.* 1996 [30] (san96), Sugimoto *et al.*[35] (sug96), SantaLucia *et al.* 2004 [6] (san04), Tanaka *et al.*[36] (tan04) were used to predict the melting temperature of 16 different not self-complementary DNA sequences from Santalucia *et al.* 1996 [30]. The prediction were done in the presence of 1 M sodium and 0.0004 M of oligomer. The line represent perfect prediction.

As illustrated by Figure 4 and Additional file 3: Figures S1 and Additional file 4: Figures S2, the accuracy of the melting temperature for DNA/DNA and RNA/RNA perfectly matching sequences has slightly improved in MELTING 4.3 and significantly improved in MELTING 5. MELTING 4.3 improvements are due to a bug fix related to the initiation parameters from Allawi and Santalucia [33] (all97) and the usage of the thermodynamic parameters provided by Santalucia and Hicks [6] (san04). MELTING 5 improves over MELTING 4.3 by computing the duplex initiation as described in each publication from which the initiation parameters originate. For instance, in the thermodynamic parameters published by Breslauer *et al.* in 1986 [34] (bre86), there are two initiation parameters: one to use when having two terminal A/T base pairs and the other to use when at least one terminal base pair is G/C. This is different from the thermodynamic parameters published by Allawi and Santalucia [33] where the initiation parameters depend on the total count of A/T and G/C terminal base pairs. In MELTING 4, all the initiation parameters relied on this formula independently from the chosen thermodynamic models. That the initiation parameters from Breslauer *et al.*[34] (bre86) were simply not taken into account explains the slight inaccuracy of the melting temperature.

**Figure 5:**
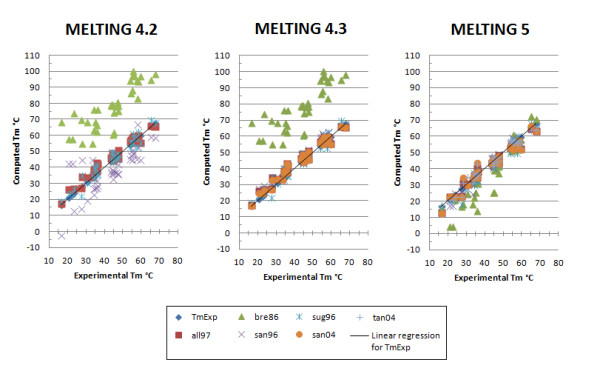
**MELTING results for DNA self complementary sequences.** The parameters sets specified by Allawi *et al.*[33] (all97), Breslauer *et al.*[34] (bre86), SantaLucia *et al.* 1996 [30] (san96), Sugimoto *et al.*[35] (sug96), SantaLucia *et al.* 2004 [6] (san04), Tanaka *et al.*[36] (tan04) were used to predict the melting temperature of 36 different self-complementary DNA sequences from Santalucia *et al.* 1996 [30]. The prediction were done in the presence of 1 M sodium and 0.0001 M of oligomer. The line represent perfect prediction.

MELTING 5 automatically sets the nucleic acid correction factor (F) appropriately for self-complementary sequences while the previous version needed user input to determine this. In addition to this, MELTING 5 uses an entropy symmetry correction term for self complementary sequences, further improving the prediction (see Figure 5 and Additional file 5: Figure S3).

The new version uses the same thermodynamic parameters for DNA single mismatches, DNA single dangling ends and inosine as the previous version(s). However, the accuracy of the predictions for duplexes containing single mismatches, single dangling ends or inosine bases has improved along with the accuracy of the perfectly matching Watson-Crick base pairs predictions. Moreover, MELTING 5 improved on these features by allowing the users to change the single mismatch, single dangling end and inosine computation by easily integrating other thermodynamic formulae and parameters. MELTING 5 also incorporates a penalty for terminal IU base pair in RNA/RNA duplexes and accepts terminal inosine pairing unlike MELTING 4.3.

The new software provides a larger choice of ion corrections (sodium, magnesium, mixed monovalent/bivalent cations) than its previous versions. Unlike the versions 4.x of the program which only proposed ion corrections determined on DNA/DNA duplexes, MELTING 5 provides specific ion corrections for DNA/DNA and RNA/RNA duplexes.

Sodium equivalence formulae allow the user to choose an approximative formula even though several ion species are present, resulting in a better approximation of the melting temperature. 18 different non self-complementary DNA sequences from Owczarzy *et al.*, 2008 [21] were used to test the accuracy of the sodium equivalence computations as implemented in MELTING 5. The Tris buffer concentration was 0.002 M (which can be equivalent to a sodium concentration of 0.001 M) and the oligomer concentration was 0.000002 M. For each sequence, five different magnesium ion concentrations were tested (0.0005 M, 0.0015 M, 0.003 M, 0.01 M, 0.02 M). The error margin was computed as follow:

(10) $$\mathrm{Err}=\left|\frac{\mathrm{Tm}(\mathrm{computed})-\mathrm{Tm}(\mathrm{experimental})}{\mathrm{Tm}(\mathrm{experimental})}\right|$$

Table 3 lists these results.

**Table 3:** MELTING error margin for sodium equivalence formulae

MELTING 4		
Approximative formula	Sodium equivalence formula	Error percentage
wet91 [4]	none	66.86
MELTING 5		
Approximative formula	Sodium equivalence formula	Error percentage
wetdna91 [4]	ahs01 [15]	3.5
wetdna91 [4]	mit96 [37]	3.6
wetdna91 [4]	pey00 [38]	3.4

Error margin in percents between experimental melting temperatures and temperatures computed with each sodium and magnesium equivalence formula implemented by MELTING.

### Current limitations of MELTING 5

MELTING is limited to publicly available thermodynamic parameters, formulae and corrections. Consequently we cannot guarantee that computations are always accurate (see the documentation of MELTING 5 for more information about the limitations of each thermodynamic models, formulae and corrections at http://www.ebi.ac.uk/compneur-srv/melting/melting5-doc/melting.html). In addition, as there is currently no algorithm to take into account the relationship between denaturing agents and ion species, the implemented formamide and DMSO corrections are independent of the ion corrections. This is biologically inaccurate and can decrease the accuracy of the predictions. However, the flexibility of the MELTING 5 code base would allow a quick integration of any new relevant research results.

### Comparison with other similar software

We have compared MELTING 5 to several other software predicting the melting temperature of nucleic acids. These are: the DINAMelt web server (http://dinamelt.bioinfo.rpi.edu/), the dnaMATE software (http://protein.bio.puc.cl/cardex/servers/dnaMATE), MeltDNA (http://sourceforge.net/projects/meltdna/), DAN (http://emboss.open-bio.org/wiki/Appdocs), MeltTemp (http://www.molgen.mpg.de/∖(∖sim∖)service/scisoft/gcg/gcg10/melttemp.html), the POLAND server (http://www.biophys.uni-duesseldorf.de/local/POLAND/poland.html), FracTM (http://www.zaik.uni-koeln.de/bioinformatik/ftpm.html) and piRNA http://compbio.cs.sfu.ca/taverna/pirna/.

Unlike most of these programs, MELTING 5 does not yet offer a web interface nor a graphical user interface. Several of them compute fuller melting profiles containing asborbance, heat capacity or other parameters, while MELTING only computes melting temperature, enthalpy and entropy.

However, only MELTING offers customisation of the following parameters: type of hybridization, thermodynamic parameters, computation methods, duplex patterns, as well as ion and denaturing agent corrections. Moreover, MELTING is the only software that allows the use of approximative formulae when several ion species are present using sodium equivalence formulae. It is also the only software which integrates parameters for nucleic acids such as inosine or locked nucleic acids, and parameters for other biochemical entities such as azobenzene. Finally, only MELTING integrates experimental evidence from all sources, eventually allowing it to handle various duplexes in various environments.

This comparison is detailed in Additional file 6: basic software structure, hybridizations, ion corrections, denaturing agent corrections, approximative formulae and polymer mode, supported duplex patterns.

## Conclusions

Two new versions of MELTING have been released. MELTING 4.3 is an extension of MELTING 4.2 and integrates inosine thermodynamic parameters for DNA and RNA sequences as well as a DNA/DNA correction for mixed monovalent/bivalent ion and magnesium ion. MELTING 5 is an extensible and flexible program which computes melting temperature for DNA, RNA, DNA/RNA and 2’-O-methyl RNA/RNA duplexes using a large choice of nearest-neighbor formulae and parameters as well as approximative formulae, and takes into account sodium, potassium, magnesium ions concentrations, Tris buffer concentrations, formamide concentration and percentages of DMSO.

MELTING 5 allows user specified customization of methods. The rationale for the development of this flexible extensible architecture is to integrate new knowledge in the thermodynamics of nucleic acids and melting temperature and to introduce a large choice of formulae, thermodynamic parameters and ion corrections. For instance, modified nucleic acids or fluorophores have become useful for certain molecular experiments and MELTING should be able to take these chemical entities into account. The extensibility of MELTING 5 allows one to easily add these new modified nucleic acids or chemical entities (like fluorophores) when thermodynamic parameters become available for them, as well as new ion species corrections. MELTING 5 implements several thermodynamic formulae which allow one to compute the enthalpy and entropy of mismatches, bulges, dangling ends, 2 to 7 CNG repeats, and will be able to integrate new thermodynamic formulae and parameters as they become available.

## Availibility and requirements

· **Project name:** MELTING

· **Web site:**http://www.ebi.ac.uk/compneur-srv/melting/

· **Project home page:**http://sourceforge.net/projects/melting/. MELTING 4.3 is also distributed by the Debian and Ubuntu projects.

· **Operating system:** Platform independent

· **Programming language:** C and a graphical interface in Perl for MELTING 4.3, Java for MELTING 5

· **Other requirements:** JRE 5 or higher for MELTING 5.

· **License:** GNU GPL version 2

## Additional files

## Competing interests

The authors declare that they have no competing interests.

## Authors’ contributions

Marine Dumousseau implemented the MELTING 4.3 extension and the MELTING 5 software. Nicolas Le Novère implemented the previous C versions of MELTING and supervised all the project. Nicolas Rodriguez helped with the implementation of the MELTING 4.3 web interface. Marine Dumousseau, Nick Juty and Nicolas Le Novère wrote the manuscript. All authors read and approved the final manuscript.

## Supplementary Material

Additional file 1 **MELTING 5 thermodynamic formulae and parameters.** This additional file lists all the patterns which can be managed by MELTING 5 and the references of each matching thermodynamic formulae and parameters. MELTING 5 cannot currently compute terminal mismatches, all mismatches must be internal. You can refer to the documentation in the MELTING 5 package or at the following page (http://www.ebi.ac.uk/compneur-srv/melting/melting5-doc/melting.html) to have more information about the MELTING 5 implementation of the thermodynamic formulae. Click here for file

Additional file 2 **Ion corrections.** This additional file lists all the ion corrections which can be applied by MELTING 5 and MELTING 4.3 as well as the references of each matching ion correction. You can refer to the documentation in the MELTING 5 package or at the following page (http://www.ebi.ac.uk/compneur-srv/melting/melting5-doc/melting.html) to have more information about the MELTING 5 implementation of the ion corrections. Click here for file

Additional file 3 **Figure S1.** MELTING results for RNA sequences. The various predictions were computed for 16 different not self-complementary RNA sequences from Xia *et al.* [[39]]. The sodium concentration was 1 M and the oligomer concentration 0.0002 M. The thermodynamic parameters with which the melting temperature was computed were Freier *et al.* [[40]] (fre86) and Xia *et al.* [[39]] (xia98). Click here for file

Additional file 4 **Figure S2.** MELTING results for DNA/RNA duplexes. The various predictions were computed for 29 different DNA/RNA duplexes from Sugimoto *et al.* [[41]]. The sodium concentration was 1 M and the oligomer concentration 0.0001 M. The thermodynamic parameters with which the melting temperature was computed were Sugimoto *et al.* [[41]] (sug95). Click here for file

Additional file 5 **Figure S3.** MELTING results for RNA self complementary sequences. The various predictions were computed for 36 different RNA self complementary sequences from Xia *et al.* (1998) [[39]]. The sodium concentration was 1 M and the oligomer concentrations 0.0001 M. The thermodynamic parameters with which the melting temperature was computed were Freier *et al.* [[40]] (fre86) and Xia *et al.* [[39]] (xia98). Click here for file

Additional file 6 **Features comparison of MELTING with similar softwares.** This additional file lists the software structure information, the supported hybridization and parameters, the ion and denaturing corrections, the approximative formulae and the supported duplex patterns of MELTING and other similar softwares. Click here for file
