# Supplementary figures and images for: MELTING, a flexible platform to predict the melting temperatures of nucleic acids
Source: BMC Bioinformatics. 2012 May 16;13:101. doi: 10.1186/1471-2105-13-101 (PMC3733425; doi:10.1186/1471-2105-13-101)

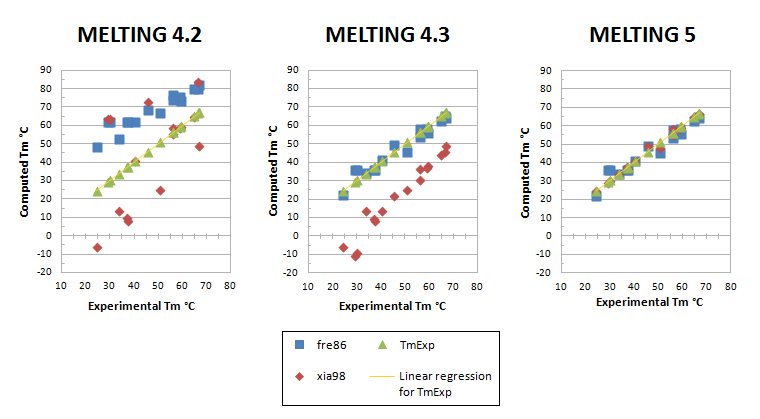

Supplement: Additional file 3 — Figure S1. MELTING results for RNA sequences. The various predictions were computed for 16 different not self-complementary RNA sequences from Xia et al. [[39]]. The sodium concentration was 1 M and the oligomer concentration 0.0002 M. The thermodynamic parameters with which the melting temperature was computed were Freier et al. [[40]] (fre86) and Xia et al. [[39]] (xia98). [file 1471-2105-13-101-S3.png]

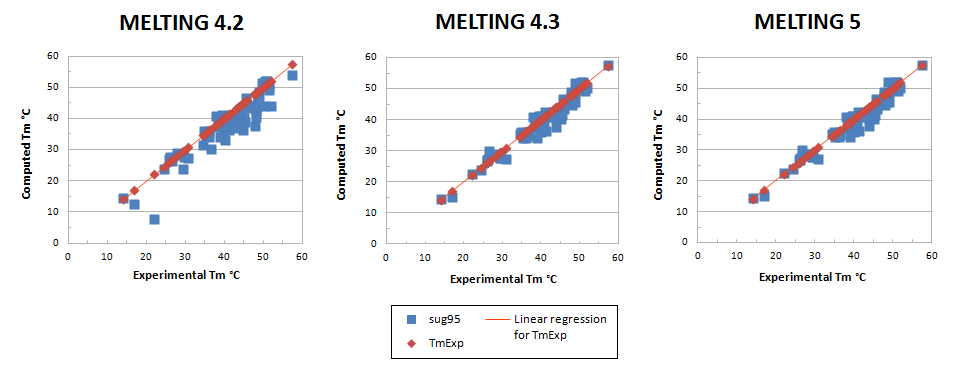

Supplement: Additional file 4 — Figure S2. MELTING results for DNA/RNA duplexes. The various predictions were computed for 29 different DNA/RNA duplexes from Sugimoto et al. [[41]]. The sodium concentration was 1 M and the oligomer concentration 0.0001 M. The thermodynamic parameters with which the melting temperature was computed were Sugimoto et al. [[41]] (sug95). [file 1471-2105-13-101-S4.png]

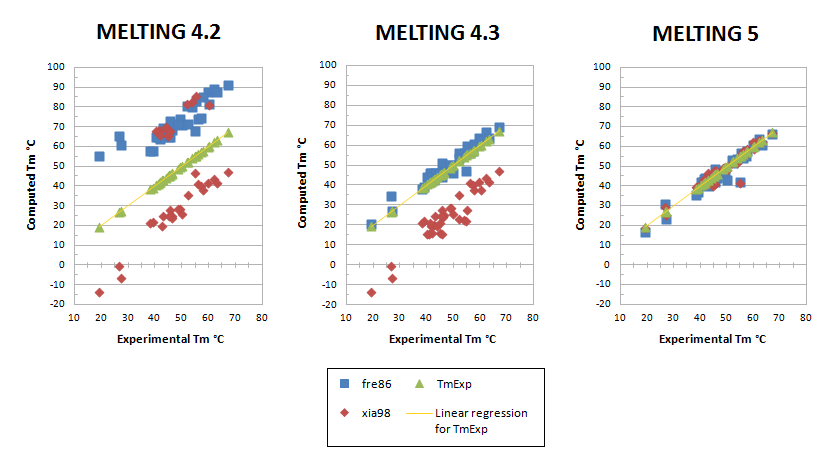

Supplement: Additional file 5 — Figure S3. MELTING results for RNA self complementary sequences. The various predictions were computed for 36 different RNA self complementary sequences from Xia et al. (1998) [[39]]. The sodium concentration was 1 M and the oligomer concentrations 0.0001 M. The thermodynamic parameters with which the melting temperature was computed were Freier et al. [[40]] (fre86) and Xia et al. [[39]] (xia98). [file 1471-2105-13-101-S5.png]
